# HCV core protein binds to gC1qR to induce A20 expression and inhibit cytokine production through MAPKs and NF-κB signaling pathways

**DOI:** 10.18632/oncotarget.9304

**Published:** 2016-05-11

**Authors:** Xiaotian Song, Zhiyan Yao, Jianling Yang, Zhengzheng Zhang, Yuqing Deng, Miao Li, Cuiqing Ma, Lijuan Yang, Xue Gao, Wenjian Li, Jianguo Liu, Lin Wei

**Affiliations:** ^1^ Department of Immunology, Hebei Medical University, Shijiazhuang, China; ^2^ Key Laboratory of Immune Mechanism and Intervention on Serious Disease in Hebei Province, Shijiazhuang, China; ^3^ Division of Infectious Diseases, Allergy and Immunology, Departments of Internal Medicine and Molecular Microbiology and Immunology, Saint Louis University School of Medicine, St. Louis, MO, USA

**Keywords:** hepatitis C virus, core protein, macrophage, negative regulator A20, gC1qR, Immunology and Microbiology Section, Immune response, Immunity

## Abstract

Hepatitis C virus (HCV) infection is characterized by a strong propensity toward chronicity. During chronic HCV infection, HCV core protein is implicated in deregulating cytokine expression that associates with chronic inflammation. A20 is known as a powerful suppressor in cytokine signaling, in this study, we explored the A20 expression in macrophages induced by HCV core protein and the involved signaling pathways. Results demonstrated that HCV core protein induced A20 expression in macrophages. Silencing A20 significantly enhanced the secretion of IL-6, IL-1β and TGF-β1, but not IL-8 and TNF. Additionally, HCV core protein interacted with gC1qR, but not TLR2, TLR3 and TLR4 in pull-down assay. Silencing gC1qR abrogated core-induced A20 expression. Furthermore, HCV core protein activated MAPK, NF-κB and PI3K/AKT pathways in macrophages. Inhibition of P38, JNK and NF-κB but not ERK and AKT activities greatly reduced the A20 expression. In conclusion, the study suggests that HCV core protein ligates gC1qR to induce A20 expression in macrophages *via* P38, JNK and NF-κB signaling pathways, which leads to a low-grade chronic inflammation during HCV infection. It represents a novel mechanism by which HCV usurps the host for persistence.

## INTRODUCTION

Hepatitis C virus (HCV) infection presents a serious public health problem worldwide [1]. Studies confirm that majority patients of HCV infection will develop chronic infection, which in turn leads to the occurrence of liver cirrhosis and hepatocellular carcinoma (HCC) [2]. The mechanism accounting for the high rate of persistent infection is that this virus has evolved one or multiple mechanisms to evade the host immunity [3–7]. Thus, the molecular mechanisms by which the virus establishes chronic infection are under intense investigation.

HCV core protein (21kDa), synthesized upon virus infection and replication, takes part in activities of virus life cycle and in assembly of virus particles. It is released from infected liver cells [8] and exhibits multiple functions to affect host immunity [9–13]. Many researchers have found that HCV core protein combining with gC1qR can inhibit the proliferation of T lymphocytes by reducing IL-2 and IL-2Rα gene transcription [14], and impair IL-12 secretion by macrophages through induction of negative regulators such as Tim-3, PD-1 and SOCS-1 [15, 16]. It can also reduce the ability of DC cells to present antigen by down-regulating the MHC-I expression [17].

Liver as the main host for HCV infection is composed of hepatocytes, liver stromal cells, and a variety of immunocytes which include DCs, macrophages, NK cells, NKT, T and B lymphocytes [18]. Macrophages and NK cells in the liver function as the first line of defense against infection similar as those in other organs [19]. Kupffer cells, traditionally denote hepatic resident macrophages, represent up to 15~20% of the total number of liver cells, and 80~90% of the total body macrophage pool [20]. Polarization of macrophages to different subtypes with distinct functions depends on the tissue microenvironment and external stimuli [21]. The previous studies in our team demonstrated that the phosphorylation of transcription factor STAT3 and the expression of membrane CD206 are up-regulated in macrophages co-cultured with HCV core protein and liver cells, which increases macrophage tendency to become M2 type macrophages [22]. M2 macrophages have been implicated in inhibiting inflammatory responses and immuno-suppression [23–26], resulting in adverse effects on HCV elimination. In other words, in the settings of persistent HCV infection, macrophage activities transformed in liver pathological microenvironment may be responsible for developing chronic HCV diseases. Macrophages can devour pathogens, meanwhile release large amounts of cytokines that impact on the strength and types of adaptive immune responses [27]. In addition, activated macrophages can also produce multiple negative regulators [28, 29]. The negative regulators contribute to inhibit further activation of inflammatory signaling pathways, thereby, protect the host from excessive immune responses [30, 31].

In this study, the aims are to explore the negative regulator A20 expression in macrophages induced by HCV core protein and the receptors and pathways involved in A20 induction. A20 (tumor necrosis factor α inducible protein 3, TNFAIP3), which is known as a powerful suppressor in cytokine signaling, can inhibit the activity of NF-κB and NF-κB-mediated inflammatory responses [32–34]. The results indicated that the interaction of HCV core protein with gC1qR could upregulate the expression of A20, which down-regulated the secretion of IL-6, IL-1β and TGF-β1 in macrophages. gC1qR, which is the receptor of the globular heads of complement C1q, is involved in the immune response to the inflammation and microbial infection [35, 36]. HCV core protein/gC1qR engagement on macrophages triggered the activation of MAPK, NF-κB and PI3K/AKT signaling pathways, among which P38, JNK and NF-κB pathways activated the A20 expression. This study reveals that HCV core protein causes a low-grade chronic infection mediated by macrophages which promotes the establishment of chronic HCV infection.

## RESULTS

### HCV core protein induces the expression of negative regulator A20 in macrophages

**Figure 1:**
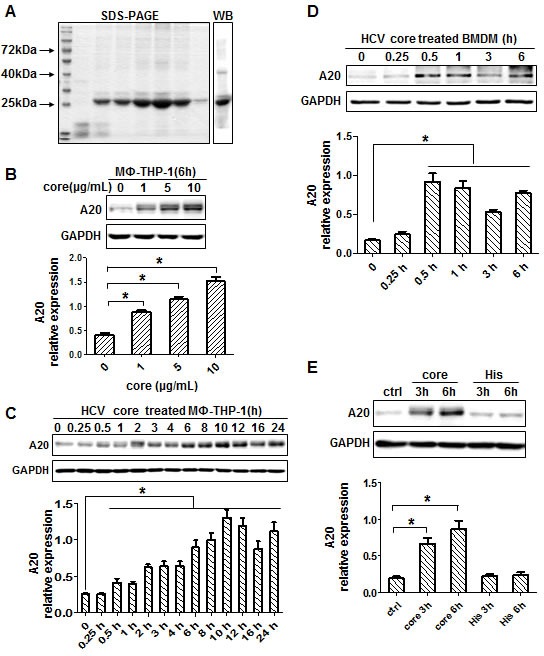
HCV core protein induces A20 expression **A.** HCV core protein was analyzed by SDS-PAGE and Western blot. **B.** A20 was upregulated in MΦ-THP-1 cells treated with different doses of core protein (1, 5, 10μg/mL). MΦ-THP-1 cells **C.** and BMDM **D.** were treated with core protein (10μg/mL) for different times, and cell lysates were analyzed by immunoblotting to examine the A20 expression. **E.** MΦ-THP-1 cells were treated with core protein and His peptide for 3 and 6 hours, and cell lysates were analyzed by immunoblotting to examine the A20 expression.

HCV core protein was successfully expressed and purified, and proved to be a single band by SDS-PAGE and Western blot (Figure 1A). The protein contained 3 EU/mL of endotoxin and was quantified as 0.5 mg/mL by Bradford method. Human monocytes (THP-1) derived macrophages (MΦ-THP-1) were generated by PMA treatment for 48 hours. To examine the expression of A20, MΦ-THP-1 cells were treated with different amounts of HCV core protein for different times. The results showed that the expression of A20 in macrophages was dose-dependently upregulated by HCV core protein (Figure 1B). Next, chose 10μg/mL core protein to treat macrophages (MΦ-THP-1), the expression of A20 began to increase at 30 minutes, and reached the peak at 10 hours (Figure 1C), while in mouse BMDM (bone marrow derived macrophage), it was induced much faster as early as 0.25 hour and peaked at 0.5 hours by core protein (Figure 1D). Meanwhile, His peptide (10μg/mL), a tag used for purification of the HCV core protein, did not induce A20 expression (Figure 1E).

### A20 inhibits the production of IL-6, IL-1β and TGF-β1 in macrophages

**Figure 2:**
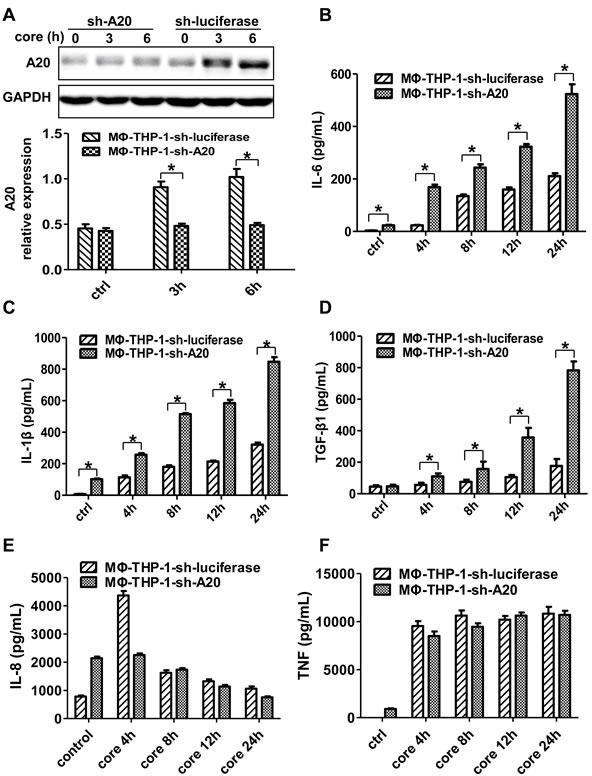
A20 negatively regulates the IL-6, IL-1β and TGF-β1 production **A.** MΦ-THP-1 cells expressing A20-specific shRNA or luciferase-specific shRNA were treated with core protein for 3 and 6 hours, cell lysates were analyzed by immunoblotting to examine the A20 expression. The secretion of IL-6 **B.**, IL-1β **C.**, TGF-β1 **D.**, IL-8 **E.** and TNF **F.** in cell culture supernatants of MΦ-THP-1 cells expressing A20-specific shRNA or luciferase-specific shRNA treated with core protein (10μg/mL) in different times were analyzed by CBA.

To determine the relationship between A20 and cytokine expression, this study used shRNA to knockdown A20 and generated A20 knockdown cells (MΦ-THP-1-sh-A20) and control cells (MΦ-THP-1-sh-luciferase). A20 was markedly suppressed in MΦ-THP-1-sh-A20 cells treated with HCV core protein (*p* < 0.05) (Figure 2A), indicating a successful knockdown of A20 by shRNA. Interestingly, the levels of IL-6, IL-1β and TGF-β1 in the supernatants of MΦ-THP-1-sh-A20 cells treated with HCV core protein were much higher than those in control cells at different time points (*p* < 0.05) (Figure 2B, 2C, 2D), demonstrating that IL-6, IL-1β and TGF-β1 were down-regulated by A20. The level of IL-8 was not changed in the supernatants of the two cell lines treated with core protein after 8h, 12h, 24h, however, after 4h, IL-8 in the supernatants of MΦ-THP-1-sh-A20 cells was lower than that in control cells (Figure 2E). The level of TNF was not changed in the supernatants of the two cell lines treated with core protein at different time points (Figure 2F). The result indicates that IL-8 and TNF are not down-regulated by A20.

### HCV core protein interacting with gC1qR induces A20 expression

**Figure 3:**
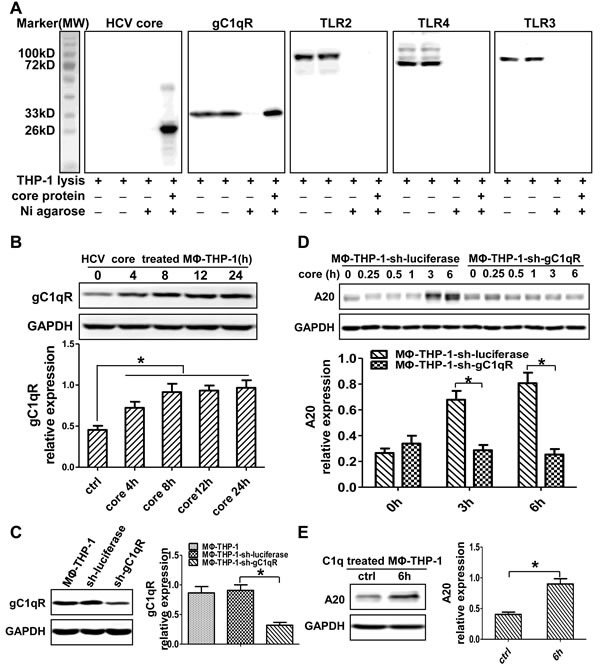
HCV core protein interacting with gC1qR induces A20 expression **A.** MΦ-THP-1 cell lysates were incubated with purified core protein coupling Ni agarose, the bound proteins were identified by immunoblotting with indicated antibodies. **B.** MΦ-THP-1 cells were treated with core protein (10μg/mL) for 4, 8, 12 and 24 hours, cell lysates were analyzed by immunoblotting to examine the gC1qR expression. **C.** Cell lysates of MΦ-THP-1 and MΦ-THP-1 cells expressing gC1qR-specific shRNA or luciferase-specific shRNA were analyzed by immunoblotting to detect the gC1qR expression. **D.** MΦ-THP-1 cells expressing gC1qR-specific shRNA or luciferase-specific shRNA were treated with core protein for indicated times. Cell lysates were analyzed by immunoblotting to examine the A20 expression. **E.** Experiment was carried out to test A20 expression in MΦ-THP-1 cells treated with C1q (70μg/mL) for 6 hours.

To determine whether core protein physically interacts with membrane receptors in MΦ-THP-1, the pull-down experiments were performed. As shown in Figure 3A, HCV core protein could interact with gC1qR but not TLR2, TLR3 and TLR4. The expression of gC1qR was upregulated in macrophages treated by HCV core protein (Figure 3B). Subsequently, knocked-down gC1qR by shRNA and established gC1qR knockdown cell line (THP-1-sh-gC1qR), the gC1qR expression in MΦ-THP-1-sh-gC1qR cells was decreased than that in control cells (MΦ-THP-1-sh-luciferase) (*p* < 0.05) (Figure 3C). A20 was also decreased in THP-1-sh-gC1qR cells treated with HCV core protein than that in control cells (*p* < 0.05) (Figure 3D). Meanwhile, A20 expression was increased in macrophages treated by C1q (a gC1qR ligand) (*p* < 0.05) (Figure 3E). These data indicate that HCV core protein induces A20 expression by interacting with gC1qR.

### JNK and P38 MAPK signaling pathways play pivotal roles in core protein/gC1qR-mediated A20 induction

**Figure 4:**
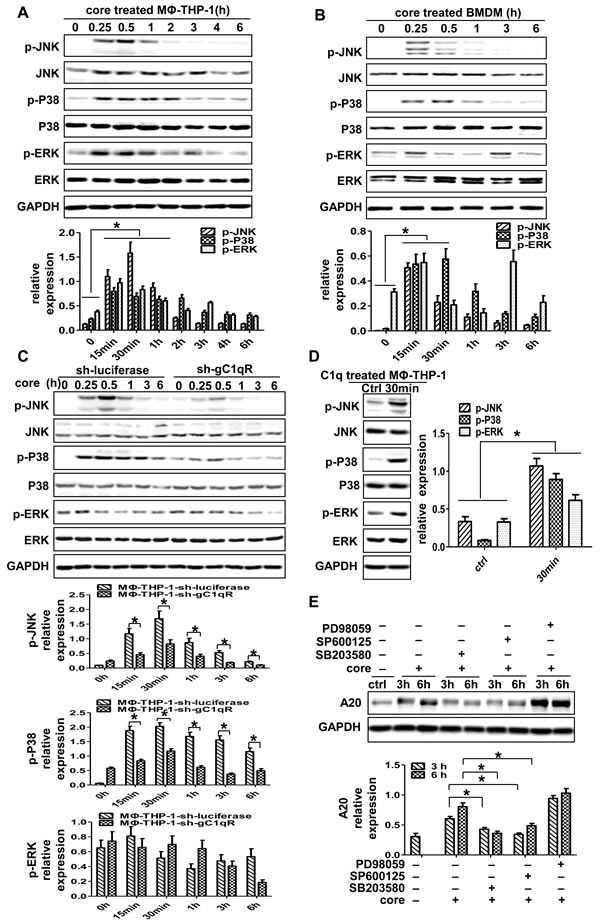
P38 and JNK signaling pathways play pivotal roles in the induction of A20 MΦ-THP-1 cells **A.**, BMDM **B.** and MΦ-THP-1 cells expressing gC1qR-specific shRNA or luciferase-specific shRNA **C.** were treated with core protein (10μg/mL) in different times, cell lysates were analyzed by immunoblotting with indicated antibodies. **D.** MΦ-THP-1 cells were treated with C1q (70μg/mL) for 30 minutes, cell lysates were analyzed by immunoblotting with indicated antibodies. **E.** MΦ-THP-1 cells were pretreated with SB203580 (30μM), SP600125 (20μM) or PD98059 (50μM) for 30 minutes respectively, then incubated with core protein for 3 and 6 hours, cell lysates were analyzed by immunoblotting to examine the A20 expression.

To identify the effect of MAPK pathways on A20 induction, whether HCV core protein could trigger the activation of MAPK pathways is questioned firstly. As shown in Figure 4A and 4B, P38, JNK and ERK were activated in MΦ-THP-1 cells and BMDM treated with HCV core protein (*p* < 0.05). Then, THP-1-sh-gC1qR cells were treated with HCV core protein, the expression of p-P38 and p-JNK were significantly lower than control cells (MΦ-THP-1-sh-luciferase) (*p* < 0.05) (Figure 4C). Furthermore, C1q, the ligand of gC1qR, was used to treat MΦ-THP-1 cells, and found that C1q could also activate P38, JNK and ERK pathways (Figure 4D). The results indicated that HCV core protein acted through gC1qR to activate P38, JNK, and ERK signaling pathways. Next, MΦ-THP-1 cells were pretreated with specific inhibitors for P38 (SB203580), JNK (SP600125), ERK (PD98059), respectively, prior to treatment with core protein, and followed by measuring A20. As shown in Figure 4E, A20 was significantly inhibited after blocking P38 and JNK pathways, but not the ERK pathway. These results suggest that P38 and JNK pathways mediate A20 expression induced by HCV core protein in macrophages.

### NF-κB signaling pathway involves in HCV core protein-mediated A20 induction

**Figure 5:**
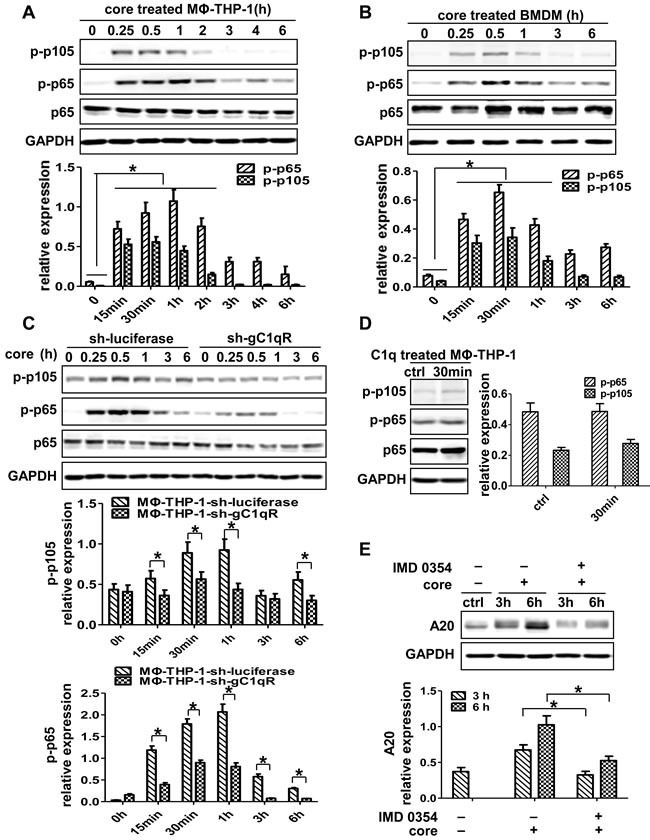
NF-κB signaling pathway plays critical role in the induction of A20 MΦ-THP-1 cells **A.**, BMDM **B.** and MΦ-THP-1 cells expressing gC1qR-specific shRNA or luciferase-specific shRNA **C.** were treated with core protein (10μg/mL) in different times, cell lysates were analyzed by immunoblotting with indicated antibodies. **D.** MΦ-THP-1 cells were treated with C1q (70μg/mL) for 30 minutes, cell lysates were analyzed by immunoblotting with indicated antibodies. **E.** MΦ-THP-1 cells were pretreated with IMD 0354 (3μM) for 30 minutes, then incubated with core protein for 3 and 6 hours, cell lysates were analyzed by immunoblotting to examine the A20 expression.

To figure out whether NF-kB involves in A20 induction, we detected the NF-kB p65 and p105 in MΦ-THP-1 and BMDM treated with HCV core protein. As shown in Figure 5A and 5B, p-NF-κB p65 and p-NF-κB p105 were upregulated in macrophages stimulated by core protein (*p* < 0.05). Furthermore, the expression of p-NF-κB p65 and p105 were measured in THP-1-sh-gC1qR cells treated with HCV core protein, the results showed significantly lower levels of p-p65 and p-p105 in gC1qR knockdown cells than control cells (Figure 5C). In contrast, unlike core protein, C1q failed to activate NF-κB pathway in MΦ-THP-1 (Figure 5D). Next, MΦ-THP-1 cells were pretreated with the inhibitor for IKKβ (IMD 0354) to block NF-κB pathway prior to treatment with core protein, and found that the expression of A20 was significantly inhibited (*p* < 0.05) (Figure 5E). These results indicate that NF-κB pathway is activated by HCV core protein and participated in A20 induction.

### PI3K/AKT pathway is not directly involved in A20 induction by HCV core protein in macrophages

**Figure 6:**
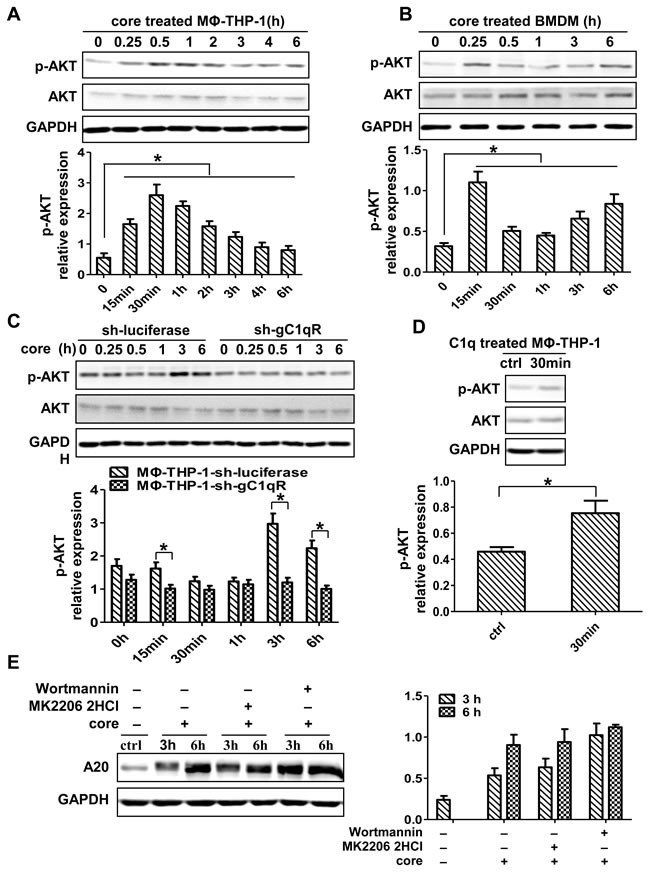
PI3K/AKT signaling pathway has no direct effect on the induction of A20 MΦ-THP-1 cells **A.**, BMDM **B.** and MΦ-THP-1 cells expressing gC1qR-specific shRNA or luciferase-specific shRNA **C.** were treated with core protein (10μg/mL) in different times, cell lysates were analyzed by immunoblotting with indicated antibodies. **D.** MΦ-THP-1 cells were treated with C1q (70μg/mL) for 30 minutes, cell lysates were analyzed by immunoblotting with indicated antibodies. **E.** MΦ-THP-1 cells were pretreated with MK 2206 2HCl (5μM) or Wortmannin (200nM) for 30 minutes respectively, then incubated with core protein for 3 and 6 hours, cell lysates were analyzed by immunoblotting to examine the A20 expression.

To define the role of PI3K/AKT pathway in core protein-mediated A20 induction, the activity of p-AKT was measured. As shown in Figure 6A and 6B, p-AKT was increased in MΦ-THP-1 and BMDM upon core protein treatment (*p* < 0.05). The expression patterns of p-AKT were different between two cells, in that it reached peak at 30 min in MΦ-THP-1 cells, whereas its expression in BMDMs had two peaks with the first peak at 15 min and the second peak at 6 h, suggesting a secondary effect on p-AKT expression at the late time point in BMDMs. HCV core protein-induced p-AKT expression was abolished in the THP-1 cells knocking-down gC1qR (Figure 6C), while C1q induced p-AKT expression in THP-1 cells by interacting with gC1qR (Figure 6D). To determine whether PI3K/AKT pathway mediates A20 induction, the PI3K and AKT pathways were blocked with Wortmaninn and MK 2206 2HCl, respectively, prior to core protein treatment, and found that the expression of A20 did no decrease after blocking PI3K/AKT pathway (Figure 6E). These results indicate that though the PI3K/AKT pathway can be activated by core protein through binding to the gC1qR on macrophages, it is not involved in core protein-induced A20 expression.

## DISCUSSION

A20, an important negative regulator for cytokine expression, one of molecules induced by TNF, was discovered in human umbilical vein endothelial cells [37]. Studies reported that A20 could protect cells from TNF-α-mediated cytotoxic effect and apoptosis [38, 39], and could also inhibit NF-κB-mediated inflammatory responses [32, 33]. This study mainly focused on the negative regulatory mechanisms induced by HCV infection in macrophages. It was showed that exogenous core protein induced the negative regulator A20 expression in macrophages. In line with our observation, Jia et al recently reported that the HCV core protein enhanced A20 mRNA expression, which contributed to inefficient M1 macrophage polarization during Hepatitis C virus infection [40]. Another report from Ma et al showed that A20 played an important role in negative regulation of mDC functions during chronic viral infection [41]. Our current study demonstrates that A20 induction in macrophages has differential effects on cytokine expression, namely inhibits IL-1β, IL-6 and TGF-b1 but not IL-8 and TNF. This differential effect leads to dysregulation of cytokine balance, which may result in a state of low grade inflammatory responses and HCV chronic infection.

To identify the mechanisms of A20 expression, the receptors and signaling pathways induced by HCV core protein were analyzed. It had been reported that HCV core protein could be a ligand combining with gC1qR to regulate the host immune responses [16, 42, 43]. In addition, Chung et al suggested that HCV core protein could induce TLR tolerance in macrophages through interacting with TLR2 [44]. However, our study showed that HCV core protein could interact with gC1qR but not TLR2, TLR3 and TLR4. In addition, the study demonstrated that exogenous HCV core protein upregulated the expression of gC1qR in macrophages, which might increase the susceptibility to HCV core protein mediated immune dysregulation. It should be noted that this study only tested the interaction between core protein and membrane receptors such as gC1qR, TLR2, TLR3 and TLR4, whether HCV core protein interacts with other membrane receptors and proteins needs to be further studied.

Furthermore, this study confirmed that interaction between HCV core protein and gC1qR triggered the activation of MAPK, NF-κB and PI3K/AKT signaling pathways in macrophages, among which P38, JNK and NF-κB pathways played major roles in the induction of A20. Meanwhile, C1q, a ligand of gC1qR, activated the MAPK and PI3K/AKT signaling pathways but not NF-κB signaling in macrophages. These results indicate that exogenous HCV core protein is mainly binding to the gC1qR, however, we could not exclude other receptors and proteins in macrophages that might be involved in A20 induction by core protein. Interestingly, gC1qR knockdown abrogated the expression of A20 induced by core protein, moreover, the expression of A20 was also induced by gC1qR ligand C1q, which further confirmed that exogenous HCV core protein interacting with gC1qR induced A20 expression.

In summary, we reveal that HCV core/gC1qR engagement triggers the activation of P38, JNK and NF-κB pathways and induces high level of A20 expression. A20 down-regulates many cytokines in macrophages, resulting in low-grade inflammation in liver that may cause a constant state of damage and repair to liver cells and hepatic stellate cells, which eventually leads to liver fibrosis and cancer [45]. Therefore, exploring the mechanisms of HCV core protein-induced A20 expression in macrophages will be favorable in understanding the HCV pathogenesis and provides a novel insight for the mechanisms of HCV chronic infection.

## MATERIALS AND METHODS

### Antibodies and reagents

The following antibodies and reagents were used in this study: anti-phospho-JNK (Cell Signaling 4668); anti-phospho-P38 (Cell Signaling 4511); anti-phospho-ERK (Cell Signaling 4370); anti-phospho-NF-κBp65 (Cell Signaling 3033); anti-phospho-NF-κBp105 (Cell Signaling 3035); anti-phospho-AKT (Ser473; Cell Signaling 4060); anti-JNK (bioworld BS3630); anti-P38 (bioworld BS3567); anti-ERK (bioworld BS1112); anti-NF-κBp65 (Cell Signaling 4764); anti-AKT (bioworld MB0052); anti-TLR2(Cell Signaling 12276); anti-TLR3 (GeneTex GTX113022); anti-TLR4 (GeneTex GTX125909); anti-A20/TNFAIP3 (Cell Signaling 5630); anti-gC1qR/p33 (Millipore MAB1161); Human C1q were obtained from Sigma (C1740); Inhibitors specific for P38 (SB203580), JNK (SP600125), ERK (PD98059), IKKβ (IMD 0354), PI3K (Wortmaninn) and AKT (MK 2206 2HCl) were obtained from selleckchem. CBA Kit was obtained from BD. Ni-NTA Agarose were obtained from QIAGEN. His peptide was obtained from Chinese Peptide Company.

### Cell lines and cell culture

The human monocytic leukemia cell line THP-1 (resource center of Peking Union Medical College Hospital, China) and HEK 293T cells (Shanghai stem cell bank) were cultured in RPMI 1640 (Gibco) containing penicillin-streptomycin (100mg/mL for each drug, Solarbio), 10mM HEPES (Solarbio), 0.05mM 2-mercaptoethanol, and 10% fetal bovine serum (Gibco) at 37°C with 5% CO_2_ in a humidified atmosphere. Before each test, monocytes (THP-1) were differentiated into macrophages (MΦ-THP-1) after treated with phorbol 12-myristate 13-acetate (PMA, 15ng/mL, multiscience) for 48h. Mouse BMDMs were isolated and cultured using standard protocols [46, 47]. Bone marrow cells from BALB/c mice were collected by flushing the femurs from six-to eight-week-old mice with phosphate buffered saline (PBS) and then cultured in RPMI 1640 (Gibco) containing 10% fetal bovine serum (BI) and macrophage-colony-stimulating factor (M-CSF) (Peprotech) at 37°C with 5% CO_2_ for 7 days. After 7 days of culturing, non-adherent cells were eliminated. Adherent cells, which were highly enriched in BMDM, were recovered for studies. All the animal procedures were vetted and approved by the Hebei Medical University Ethical Committee for Animal Experimentation.

### Expression and purification of HCV core protein and pull-down assay

HCV core protein was expressed and purified as previously described [22]. This fused protein was analyzed by SDS-PAGE and Western blot with antibody (mouse anti HCV core monoclonal antibody, Santa Cruz sc-57800). HCV core protein was treated with Endotoxin Removal Kit to remove endotoxin, and then detected endotoxin in Limulus reagents assay. It was quantified by Bradford method before used in cell experiments. Pull-down assay was conducted by incubating cell lysates with purified core protein coupling Ni agarose at 4°C overnight. Agarose was washed and examined by Western blot.

### ShRNA knockdown of gC1qR or A20 and generation of cell lines

Short hairpin RNA (shRNA) expressing stable gC1qR, A20 and luciferase transformant were generated by infecting the cells with lentiviral expressing specific shRNA in pSIF1-H1-copGFP vector (System Biosciences). The sequences used in the shRNA targeting gC1qR, A20, and luciferase (the control shRNA) were as follows: 5′-GCTGAGAGTGACATCTTCTCT-3′, 5′-GGATCTGCAGTACTTGCTTCA-3′, and 5′-CTTACGCTGAGTACTTCGA-3′. Western blot was performed to determine the knockdown efficiency.

### Quantification of cytokine production by cytometric bead assay

Cytometric Bead Assay (CBA) was used to detect the levels of cytokines (TNF, IL-8, IL-6, IL-1β and TGF-β1) in cell culture supernatants of MΦ-THP-1-sh-gC1qR and MΦ-THP-1-sh-luciferase cells treated with HCV core protein (10μg/mL) for indicated times. The cells treated with PBS alone served as control groups. Data were acquired using a FACS calibur flow cytometer. The results were based on standard concentration curves and expressed as pg/mL.

### Western blot assay

MΦ-THP-1 cells were stimulated with HCV core protein (10μg/mL) for indicated times, and were lysed by Protein Extraction Reagent supplemented with PMSF and phosphatase inhibitors. Protein concentrations of the whole cell lysates were measured by Nanodrop. Samples were separated by 12% SDS-PAGE, and transferred onto the PVDF membrane, and then blotted with indicated antibodies. Immunoblot signals were quantified by densitometry using image J. The relative expression of phospho-JNK, phospho-P38, phospho-ERK, phospho-NF-κB p65, phospho-NF-κB p105 and phospho-AKT, were calculated as: the intensity of phospho-protein/the intensity of identical total protein. The relative expression of A20 was calculated as: intensity of A20/the intensity of GAPDH.

### Statistical analysis

The experiments described in this study were performed in triplicate. Results are expressed as mean ± standard deviation. Differences between variables were tested for significance using the Student's t test. Statistical analyses for multiple comparisons were conducted using the one-way analysis of variance (ANOVA) function. If data were not normally distributed, before applying ANOVA they were transformed in rank. All analyses mentioned above were fulfilled using the SPSS version 16.0 software program. A value of *p* < 0.05 was considered significant.
